# Supplementary material for: A Morphometric Approach to Understand Prokaryoplankton: A Study in the Sicily Channel (Central Mediterranean Sea)
Source: Microorganisms. 2023 Apr 13;11(4):1019. doi: 10.3390/microorganisms11041019 (PMC10142791; doi:10.3390/microorganisms11041019)
Supplement: Supplementary file 1 [file microorganisms-11-01019-s001.zip › Table S1.pdf]

Table S1. Acronyms of the studied parameters

T°C	temperature
S	salinity
DO	dissolved oxygen
DEN	density
FLUO	fluorescence
NO_3	nitrates
PO_4	phosphates
SiO_4	silicates
Chl *a*	chlorophyll *a*
PA	prokaryotic abundance
PB	prokaryotic biomass
VOL	cell volume
CCC	cell carbon content
VA	virus-like particle abundance
VPR	virus to prokaryote ratio
