# Supplementary material for: A Morphometric Approach to Understand Prokaryoplankton: A Study in the Sicily Channel (Central Mediterranean Sea)
Source: Microorganisms. 2023 Apr 13;11(4):1019. doi: 10.3390/microorganisms11041019 (PMC10142791; doi:10.3390/microorganisms11041019)
Supplement: Supplementary file 1 [file microorganisms-11-01019-s001.zip › Table S2.pdf]

Table S2. Ranges of variation, means and standard deviations of prokaryotic cell volume (VOL), cell carbon content (CCC), prokaryotic abundance (PA), prokaryotic biomass (PB) and virus –like particle abundance (VA).

	VOL	*CCC*	*PA*	*PB*	*VA*
	μm^3	fg C $cell^{-1}$	cell x 10^6 ml^{-1}	μg C l^{-1}	VLP x 10^5 ml^{-1}
BANSIC-2012					
min	0.017	0.6	0.55	24.9	0.42
max	2.12	416,0	1.95	113.08	2.83
mean	0.17	45.6	1.21	54.45	1.68
sd	0.156	35.1	.40	20.65	0.72
n	4573	4573	29	29	29
NOVESAR-2013					
min	0.012	4.7	0.25	4.8	n.d.
max	0.536	127.3	1.06	14.38	n.d.
mean	0.06	18.7	0.51	8.51	n.d.
sd	0.052	13.4	0.23	3.34	n.d.
n	1344	1344	14	14	
BANSIC-2013					
min	0.005	2.3	0.63	12.09	0.18
max	2.42	467,0	1.76	62.68	1.57
mean	0.103	29.6	1.09	32.86	0.74
sd	0.118	26.8	0.31	14.95	0.31
n	2852	2852	16	16	16
