# Supplementary material for: A Morphometric Approach to Understand Prokaryoplankton: A Study in the Sicily Channel (Central Mediterranean Sea)
Source: Microorganisms. 2023 Apr 13;11(4):1019. doi: 10.3390/microorganisms11041019 (PMC10142791; doi:10.3390/microorganisms11041019)
Supplement: Supplementary file 1 [file microorganisms-11-01019-s001.zip › Table S3.pdf]

Table S3. Average Cell Carbon Content (CCC) determined for each station and depth.

BANSIC 2012

Station	Depth	fg C $cell^{-1}$
**22**	1	65.3
	25	53.7
	45	58.1
	56	47.1
**143**	1	56.0
	25	36.9
	50	46.4
	70	55.9
	100	57.7
**137**	1	52.5
	25	48.8
	60	47.9
	70	48.7
	83	35.2
**188**	2	56.5
	25	50.8
	50	42.2
	70	37.2
	89	38.0
**302**	1	42.0
	25	35.1
	50	38.0
	70	37.1
	102	36.8
**641**	1	51.5
	25	36.7
	50	32.9
	70	34.6
	95	34.2

NOVESAR 2013

Station	Depth	fg C $cell^{-1}$
**22**	1	16.8
	25	18.2
	45	18.5
	56	18.4
**137**	10	15.0
	25	13.6
	60	18.5
	70	16.8
	87	16.9
**188**	1	20.9
	25	17.8
	50	24.3
	70	23.4
	91	22.3

BANSIC 2013

Station	Depth	fg C $cell^{-1}$
**22**	0	48.2
	9	51.2
	25	42.3
	65	35.6
**140**	0	24.5
	9	26.4
	25	23.7
	70	22.9
**641**	0	20.9
	15	22.1
	25	31.0
	75	24.9
**302**	0	31.0
	8	25.1
	25	24.0
	68	28.7
